# Antibiotic resistance pattern of the strains isolated in urinary tract infections in the last semester in the military hospital

**DOI:** 10.1186/1471-2334-13-S1-P33

**Published:** 2013-12-16

**Authors:** Simona Costin, Michaela Oana, Mihai Feier, Asiza Olteanu

**Affiliations:** 1 “Dr. Constantin Papilian” Emergency Military Hospital, Cluj-Napoca, Romania

## Background

In the last years the antibiotic resistance is increasing, and self-medication is no longer the only reason. The study was designed to compare community urinary tract infections (UTI) and hospitalized patients’ UTI.

## Method

We performed a retrospective study of the resistance pattern of the main strains isolated from the hospitalized patients admitted to the Emergency Military Hospital in Cluj-Napoca between 01 January – 30 June 2013, and from the urine sample of the ambulatory patients, during the same period. For antibiotic susceptibility we used the semiautomatic VITEK 2 system and the diffusimetric method. We studied demographic characteristics, in correlation with microbiological data.

## Results

For the hospitalized patients: from 317 samples, 209 were positive, in which we isolated *E coli*=79%, *Enterobacter*=9.5%, *Proteus* spp=5%, *Klebsiella*=5%, *Enterococcus* spp, *Serratia* spp, *Staphylococcus*=1.5%. The *E coli* sensitivity to antimicrobial drugs was found to be as follows: ampicillin=30%, cefepime=50%, norfloxacin=62%, ofloxacin, ciprofloxacin=68%, biseptol=63%, amoxicillin+clavulanic acid=70%, ceftazidime=85%, amikacin=98%, nitrofurantoin and carbapenem=100%.

For the ambulatory patients: from 605 urinary samples, 318 were positive, in which we isolated: *E coli*=72.5%, *Enterobacter*=12.5%, *Proteus*=10%-mainly nosocomial, after prostatectomy or urinary catheter, *Klebsiella*, *Enterococcus*, *Citrobacter*=5%.

## Conclusion

Urine culture is a routine microbiological investigation, even when not indicated. The treatment of asymptomatic bacteriuria is a controversial issue, especially for ambulatory patients. *E coli* remains the most common strain isolated in urine cultures, and the antimicrobial stewardship is necessary.

